# 

**Published:** 1896-01

**Authors:** 


### ILLUSTRATIONS.

| | PAGE |
|---|---|
| Actinomycosis of the Inferior Maxilla | 726–727 |
| Fracture of the Trapezium | 303 |
| Ingenious and Useful Device | 478 |
| Intestinal Obstruction in the Dog | 11 |
| Œsophageal Obstruction | 131 |
| Traumatic Ventral Hernia | 301 |

MARRIED.—On Wednesday, December 11, 1895, by the Rev. S. A. Zeigenfuss, Dr. Adwam W. Ormiston to Miss Carrie Atkins Wilson, both of Germantown, Philadelphia.

Secretary.

---

### PERSONAL.

Veterinarian J. Massie, of Kingston, Canada, enjoys the distinction of ranking as captain in the Royal Canadian Artillery.

Veterinarian J. B. Heaton takes an active interest in agriculture in his field of labor. He fills the onerous position of Secretary to the Bloomfield County Fair, Indiana.

Dr. Otto Noack, of Reading, Pa., has removed his office to 529 Cherry Street, formerly occupied by the late Dr. W. U. Custer.

Dr. A. S. Wheeler, of New Orleans, La., has returned from his trip abroad.

Dr. Austin Peters, of Massachusetts, who has been recognized as a commissioned officer in the Bay State Troop, takes a deep interest in the battle for recognition with rank in the U. S. Army, and will be a strong arm of support to the committee this year.

Veterinarian W. L. Burt, of Providence, R. I., has been visiting Illinois and Indiana for the purpose of inspecting horses.

Mr. Harold Sorby, manager of the Pasteur Anthrax Vaccine Co., has removed his headquarters to 315 Rialto Building, Chicago, Ill., where a more central location will afford quicker means of covering the entire country. A supply depot will be retained in New York for the accommodation of Eastern patrons.

Mr. David Roberge, author of a text-book on the *Foot of the Horse*, is giving a course of lectures on "Shoeing" at the New York College of Veterinary Surgeons.

Drs. C. B. Robinson and H. W. Atchison aided very materially the Army Legislative Committee at the opening of its battle in Washington in December.

General Nelson A. Miles kindly accorded the Army Legislative Committee a hearing and gave them much encouragement in the movement.

Dr. W. E. A. Wyman, of Greenville, S. C., a graduate of the New York College of Veterinary Surgeons, has received the appointment of Professor of Veterinary Science at the Clemson Agricultural College, South Carolina.

Dr. Cecil French, of Washington, D. C., spent a part of his Christmas holidays at the seat of his college days, Montreal, Canada.

J. Hugo Reed, V. S., graduate of the Ontario Veterinary College, fills the Chair of Veterinary Science at the Ontario Agricultural College and Experimental Farm at Guelph.

Dr. R. S. Huidekoper filled the rôle of veterinarian to the stock-show at Madison Square Garden in November.

Veterinarians should be beware of one styling himself Dr. Wilson Carriere, of New York City, who is visiting from city to city and calling upon prominent veterinarians for loans of money to enable him to reach his home, when immediate reimbursement will follow, but which fails to follow and all communications are ignored.

Veterinarian H. T. Foote, of New Rochelle, N. Y., will officiate as one of the judges at the New York Kennel Show in February next.

Dr. J. C. Michener addressed the Farmers' Institute at Pottstown on December 18th on the "Health of Farm Animals."

Dr. Frank S. Billings, in the December 20th issue of *Turf, Field, and Farm*, reviews the history of veterinary education in America, and comments upon the relative worth of the two- and three-term schools. He demands a more practical veterinarian.

